# A Multilevel Deep Learning Model for Automated Brain Tumor Segmentation Using Magnetic Resonance Images

**DOI:** 10.3390/diagnostics16142259

**Published:** 2026-07-20

**Authors:** Aneesh S. Perumprath, Kulandairaj Martin Sagayam, Syed Immamul Ansarullah, Shafat Khan, Sami Alshmrany, Farhan Amin, Isabel de la Torre Díez, Mirtha Silvana Garat de Marin, Eduardo Silva Alvarado

**Affiliations:** 1 Department of ECE, Karunya Institute of Technology and Sciences, Coimbatore 641114, India; aneeshsamuel@gmail.com (A.S.P.); martinsagayam.k@gmail.com (K.M.S.); 2 Department of Computer Applications, Govt. Degree College Sumbal, Bandipora 193502, India; syedansr@gmail.com; 3 Department of Computer Science, College of Computer Science, King Khalid University, Abha 61421, Saudi Arabia; sbkhan@kku.edu.sa; 4 Faculty of Computer and Information Systems, Islamic University of Madinah, Madinah 42351, Saudi Arabia; s.alshmrany@iu.edu.sa; 5 School of Computer Science and Engineering, Yeungnam University, Gyeongsan 38541, Republic of Korea; 6 eHealth and Telemedicine Group, University of Valladolid, 47011 Valladolid, Spain; isator@uva.es; 7 Department of Projects, Universidad Internacional Iberoamericana, Arecibo, PR 00613, USA; silvana.marin@unic.co.ao; 8 Department of Projects, Universidad Europea del Atlántico, Isabel Torres 21, 39011 Santander, Spain; eduardo.silva@uneatlantico.es

**Keywords:** deep learning, brain, segmentation, detection, multilevel architecture-based modified U-Net

## Abstract

**Background/Objectives:** Brain tumor segmentation from magnetic resonance imaging (MRI) plays an important role in clinical assessment and treatment planning. However, accurate segmentation remains challenging because of the complex anatomical structure of the brain, variations in tumor size and shape, and the imbalance between tumor and non-tumor regions in MRI datasets. These challenges highlight the need for reliable automated segmentation methods. **Methods:** This study proposes a multilevel deep learning model for automated brain tumor segmentation using MRI images. The BraTS dataset was used for model development and evaluation. To address class imbalance, a modified Synthetic Minority Oversampling Technique (SMOTE) was incorporated during preprocessing. A Multilevel Architecture-Based Modified U-Net was then employed to learn multiscale spatial features and generate pixel-wise tumor segmentation. The proposed framework was evaluated using the Dice coefficient, Jaccard coefficient, Matthews Correlation Coefficient (MCC), and accuracy. **Results:** The experimental results demonstrate that the proposed model consistently outperformed the Berkeley Wavelet Transform (BWT)-based method and the conventional U-Net across different tumor grades. Higher Dice, Jaccard, and MCC values indicate improved agreement between the predicted segmentation and the expert-annotated ground truth masks, demonstrating more accurate and consistent tumor delineation. **Conclusions:** The proposed multilevel deep learning model provides an effective framework for automated brain tumor segmentation from MRI images. By combining imbalance-aware preprocessing with a lightweight Modified U-Net architecture, the proposed method improves segmentation performance while maintaining a relatively simple network design. Future work will focus on validating the proposed framework using external clinical datasets and comparing it with recent state-of-the-art segmentation models.

## 1. Introduction

Brain tumors are serious neurological disorders that can affect individuals of different age groups and may lead to severe health complications if not accurately assessed and treated. Magnetic resonance imaging (MRI) is widely used for brain tumor analysis because it provides high-resolution soft-tissue contrast and enables the visualization of tumor regions using different imaging modalities, such as T1-weighted, contrast-enhanced T1-weighted, T2-weighted, and fluid-attenuated inversion recovery images [1,2]. However, accurate brain tumor segmentation from MRI remains challenging because of the complex anatomical structure of the brain, heterogeneous tumor appearance, irregular tumor boundaries, low contrast between tumor and normal tissues, and the presence of imaging noise [1,2]. Brain tumor segmentation is an important step in computer-aided medical image analysis. It aims to delineate tumor regions from MRI scans and can support clinical assessment, treatment planning, and disease monitoring. Manual tumor segmentation by radiologists is time-consuming and subjective and may vary between experts, especially when tumor boundaries are unclear or tumor regions are small and irregular. Therefore, automated segmentation methods are needed to improve consistency, reduce manual workload, and provide reliable tumor region delineation [3].

Deep learning methods, particularly U-Net-based architectures, have shown strong performance in medical image segmentation. The original U-Net uses an encoder–decoder structure with skip connections to combine spatial and semantic information, making it suitable for biomedical segmentation tasks [3]. Several extensions of U-Net have also been proposed, including Attention U-Net, U-Net++, nnU-Net, and transformer-based architectures, to improve feature extraction, contextual representation, and segmentation accuracy [4,5,6]. Although these methods can achieve strong performance, many of them increase architectural complexity by adding attention gates, dense skip connections, self-configuring modules, transformer blocks, or multiple encoder–decoder branches. Such complexity may increase training difficulty and computational cost, particularly when datasets are imbalanced or when small tumor regions must be accurately segmented.

Problem Statement: The main problem addressed in this study is the accurate segmentation of brain tumor regions from MRI images under class-imbalanced conditions.

Aims of This Research: The aim of this work is to develop an automated brain tumor segmentation framework using a Multilevel Architecture-Based Modified U-Net. In this study, tumor detection refers only to the localization of tumor regions before segmentation. The proposed framework focuses on improving tumor region delineation by combining imbalance-aware preprocessing with a lightweight multilevel deep learning architecture.

Motivation of This Research: The motivation of this study is to develop a lightweight and reliable framework for automated brain tumor segmentation from MRI images, since many existing deep learning approaches improve accuracy by increasing architectural complexity through attention modules, transformer blocks, or multiple encoder–decoder branches. The proposed Multilevel Architecture-Based Modified U-Net addresses this issue by combining modified SMOTE-based preprocessing to reduce class imbalance, multilevel feature learning to capture both shallow spatial details and deeper semantic tumor information, and a simplified U-Net structure to reduce unnecessary model complexity.

Benefits of our Research: The key benefit of the proposed method is its ability to improve tumor boundary delineation and segmentation consistency while maintaining a relatively simple network design. The main contribution of this work is the integration of imbalance-aware preprocessing, multilevel feature extraction, and a lightweight Modified U-Net architecture for accurate MRI-based brain tumor segmentation using the BraTS dataset.

Problems In State-of-the-Art Research: Although numerous deep learning methods have been proposed for brain tumor segmentation, several challenges remain. Most existing approaches improve segmentation performance by incorporating attention mechanisms, residual connections, transformer modules, or multiple encoder–decoder branches, which increase model complexity and computational requirements. In addition, limited attention has been given to simultaneously addressing class imbalance and multilevel feature representation within a lightweight segmentation framework. To bridge this gap, this study proposes a Multilevel Architecture-Based Modified U-Net that integrates modified SMOTE-based preprocessing, multilevel feature learning, and a simplified encoder–decoder architecture. The modified SMOTE reduces the impact of class imbalance during training, while the multilevel architecture captures both low-level spatial details and high-level semantic features for accurate tumor boundary delineation. By reducing the number of encoding and decoding stages compared with the conventional U-Net, the proposed framework maintains a relatively simple network structure while improving segmentation performance. Therefore, the novelty of this research lies in the integration of imbalance-aware preprocessing, multilevel feature learning, and a lightweight Modified U-Net architecture within a unified framework for automated brain tumor segmentation from MRI images.

Our Key Contributions: The main contributions of this study are summarized as follows. First, a Multilevel Architecture-Based Modified U-Net is proposed for automated brain tumor segmentation from MRI images. Second, modified SMOTE-based preprocessing is incorporated to reduce the influence of class imbalance during training. Third, a multilevel feature learning strategy is used to combine shallow spatial features with deeper semantic tumor information. Fourth, a simplified U-Net architecture is designed by reducing unnecessary encoding and decoding stages while preserving segmentation performance. Finally, the proposed framework is evaluated on the BraTS dataset using the Dice coefficient, Jaccard coefficient, Matthews Correlation Coefficient, and accuracy, and its performance is compared with BWT and conventional U-Net models.

The rest of this paper is organized as follows. In Section 2, we discuss related work. The key deep learning techniques that have been used for medical image analysis are discussed in this section. In Section 3, we present our proposed work. Section 4 presents the experimental results. Section 5 presents the discussion of this research. In this section, we present key findings. Finally, Section 6 presents the conclusion of this research.

## 2. Literature Review

A crucial step in computer-aided diagnosis techniques is the identification of brain tumors, which is followed by segmentation. Several factors are involved in segmentation processes. Using deep neural networks, a unique method was presented in [7] in an attempt to separate brain pictures from 2D MRI data. The model comprised an encoder and decoder for recognizing tumors, which are normally delimited by medical professionals, as well as a preset data augmentation technique. Because low-grade gliomas were somewhat more difficult to identify using conventional methods, they are regarded as more difficult to treat than other types of malignant or tumorous cells. The authors stated that automated low-grade glioma detection and localization methods were used by the model. The Znet framework made use of a deep learning architecture, and the pixel accuracy method was used to test the strategy. Semantic segmentation was used to solve the problem of class imbalance. Ground truth values were derived from background variables and frequently substituted for falsely high pixel values. A methodology for brain tumor segmentation using convolutional neural networks on human brain MRI images was defined by [8]. The method comprised both the inference required for evaluations and the MRI scans for training. An approach to cross-modal distillation was presented in order to overcome the incapacity of current state-of-the-art methods to indicate accuracy in real-time scenarios. The multiple sequence information present in the MRI images typically required an improved CNN architecture to address the drawbacks of the single-sequence CNN model. The cross-distillation approach increased segmentation quality when compared to that of a single-sequence CNN model, according to the investigative results of the BRATS 2018 dataset, which was used to evaluate the models. Cross-distillation was shown to enhance the theoretical working model of a single-sequence convolutional neural network using multiple sequential information from MRI scans. The next method was presented as a multitasking method taking into account the element of many modalities in MRI pictures [9]. This method was described as an automated methodology using segmentation fusion, in which the task of reconstructing images with numerous characteristics fusing together was applied to a variational encoder. In order to derive features from several modalities, a regularization technique known as segmentation fusion was implemented. By comprehending the various features from the input photos, fusion was meant to improve the feature engineering process. During training, the multitasking model’s uncertainty-based strategy was aligned with the model’s adoption of weight loss. The benchmark dataset BRATS 2020 was taken into consideration to assess the model using real-time scenarios. According to the test results, the multitasking model outperformed the VAE model in terms of operations and segmentation accuracy. Using a learning and branching strategy [10], an HMRNet approach with a multiscaling feature was used to accommodate the high resolution of images. In order to track the process of delineating the Clinical Target Volume, the model aimed to segment the images of Anatomical brain Barriers to Cancer. With the primary focus being on scales, contextual information on the anatomical features of the human brain was obtained and preserved. Better segmentation is made possible by the ability to transform the characteristics into spatial attention maps through bidirectional feature calibration. It was determined that brain barriers come in a variety of sizes and forms, making segmentation challenging. The aforementioned method enhanced monodirectional and bidirectional features by improving structural information with high-resolution photos and by increasing the attention span through calibration. The MICCAI dataset, which is only available to ABCs, was used by the model. Combining Long Short-Term Memory-based convolutional neural networks with a hepatocellular carcinoma segmentation technique yielded a four-dimensional deep learning solution. The method used in [11] took into account liver tumors that were detected by MRI scans and for which contrast enhancement techniques, specifically Dynamic Contrast Enhancement, were employed. Each contrast-enhanced image’s three-dimensional attributes were extracted and sent to a 4-layered LSTM network so it could learn about the characteristics of the input images. The model effectively separated the tumors that are present in the liver region and improved the features of hepatocellular carcinoma. The model was shown to perform better than its predecessor, the 3D UNet and nnU-Net architectures, based on comparisons of the results. Additionally, the model stressed how crucial contrast enhancements are. The human brain is a multifaceted organ made up of several hemispheres, tissues, and cells. Due to overlapping features, medical imaging technologies usually have difficulty clearly identifying separate regions. Medical imaging equipment can record the axial, coronal, and sagittal views of a human brain because different perspectives of the brain are taken into consideration for a clearer view of a particular region. Furthermore, the segmentation and classification processes depend heavily on the features encoded in the brain images. In order to improve segmentation results, ref. [12] suggested a deep learning model that combined a dynamic fusion technique. In [13] the authors proposed MRI-based brain tumor prediction using a convolutional neural network framework. In this research, the authors presented a CNN-based model for automated brain tumor identification through magnetic resonance imaging (MRI). The proposed model achieved better generalization capability through preprocessed imaging that involved normalization followed by segmentation. Optimization was carried out using hyperparameter tuning, early stopping and model checkpointing. In [14], the authors used a deep learning-based framework for the detection of brain tumors. They also performed classification. In [15], the authors used transfer learning models for classification and the detection of brain tumors. In [16], the authors presented a machine learning-based approach and a statistical model for brain tumor detection. In [17], the authors introduced an effective approach for identifying brain lesions in magnetic resonance imaging (MRI) images, minimizing dependence on manual intervention. The proposed method improves image clarity by combining guided filtering techniques with anisotropic Gaussian side windows (AGSWs). In [18], the authors presented the development and evaluation of Artificial Intelligence (AI) models for the automatic the classification of brain tumors in magnetic resonance imaging (MRI) scans. In [19], the authors presented an interpretable deep learning approach for brain tumor classification using a Bangladeshi brain MRI dataset. The proposed pipeline combines image preprocessing and feature enhancement methods, and then it trains a series of squeeze-and-excitation (SE)-enhanced convolutional neural networks such as VGG19, DenseNet201, MobileNetV3-Large, InceptionV3, and EfficientNetB3. In [20], 3D MRI reconstruction and brain tumor diagnosis using deep learning with explainable AI were presented.

## 3. Proposed Model

In this section, we explain our proposed model.

**Figure 1:**
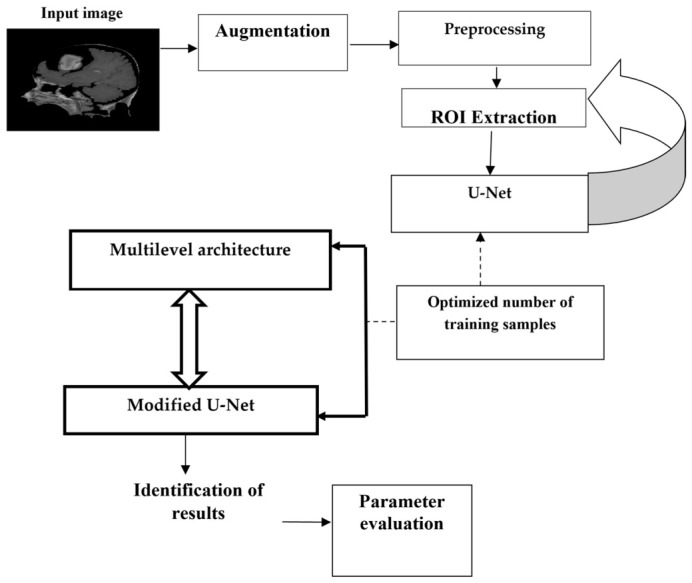
Basic flow of our proposed model.

Figure 1 shows the basic flow of the proposed work. In this diagram, we can see that the initial data came from the MRI dataset in the data acquisition phase. Then augmentation was performed prior to processing. The complete description is given below.

### 3.1. Data Acquisition

**Figure 2:**
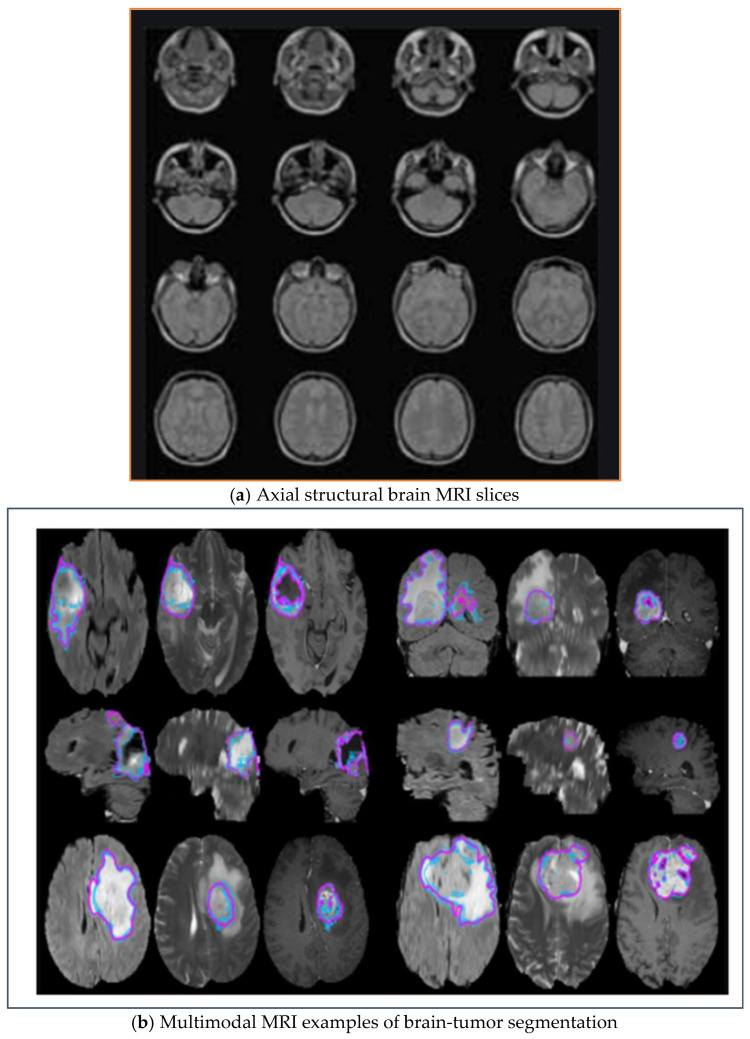
BraTS dataset.

The Benchmark for Multimodal Brain Tumor Image Segmentation (BRATS) was used in this study [14]. In the research community, the BraTS dataset is widely used as a benchmark dataset for testing BT detection methods. The experiments were conducted using the Benchmark for Multimodal Brain Tumor Image Segmentation (BraTS) dataset. The dataset includes multimodal MRI scans, namely T1-weighted, contrast-enhanced T1-weighted (T1ce), T2-weighted, and fluid-attenuated inversion recovery (FLAIR) images, together with expert-annotated tumor segmentation masks. In this study, 893 annotated images were used for model training, and 223 annotated images were used for testing. The dataset was therefore evaluated using a fixed training–testing partition. Cross-validation was not employed. To address the class imbalance among tumor grades, the modified SMOTE algorithm was applied during preprocessing. Apart from SMOTE-based balancing, no additional augmentation techniques were used in the present study. Figure 2 displays photos from the BraTS dataset. Figure 2a shows the representative axial structural brain MRI slices arranged sequentially from the inferior skull base at the upper left to the superior cerebral convexity at the lower right. Figure 2b shows the multimodal MRI examples of brain-tumor segmentation in axial, coronal, and sagittal planes. The cyan and magenta contours indicate two overlapping lesion delineations.

Several image grades are chosen here. Over 50% of individuals are affected by grade 1 or grade 2 brain tumors. In contrast, fewer people are impacted by grade 3 and grade 4 brain tumors. This is when the issue of data balancing appears. The SMOTE algorithm is utilized in this work to get around this.

### 3.2. Preprocessing

The challenge of classifying data when there is an uneven distribution of classes is known as imbalanced data classification. Many strategies have been proposed to address the imbalanced data categorization problem. Still, the integrated techniques show good performance on this difficult classification task. As a result, creating an integrated strategy is essential to enhancing the effectiveness of imbalanced data classification. The preprocessing techniques efficiently manage the unbalanced data and prepare it for categorization. The Synthetic Minority Oversampling Technique (SMOTE) is a proficient preprocessing method that produces balanced data from imbalanced content. In this study, a modified SMOTE was used only as a feature-level/sample-balancing strategy and was not applied directly to raw MRI pixels or segmentation masks. After preprocessing and feature representation, minority-grade samples were oversampled by interpolating between the feature vectors of nearest-neighbor samples belonging to the same minority class. Therefore, the method did not generate completely new anatomical MRI slices or artificial tumor masks. The purpose of this step was to reduce grade-level imbalance in the training data and prevent the model from being biased toward majority tumor classes. To reduce the risk of unrealistic synthetic samples, interpolation was restricted to nearest neighbors within the same tumor grade class, and the generated samples were used only for model training. The validation and testing stages were performed using the original MRI images and their expert-annotated masks. Nevertheless, we acknowledge that SMOTE has limitations for image segmentation because it was originally developed for tabular data. Therefore, future studies will investigate image domain augmentation methods, including rotation, intensity perturbation, elastic deformation, and generative models, to further improve anatomical realism and segmentation robustness. The computational complexity is significant, which has an impact on overall classification performance even though numerous classification algorithms have been used to classify this preprocessed data [15].

**Figure 3:**
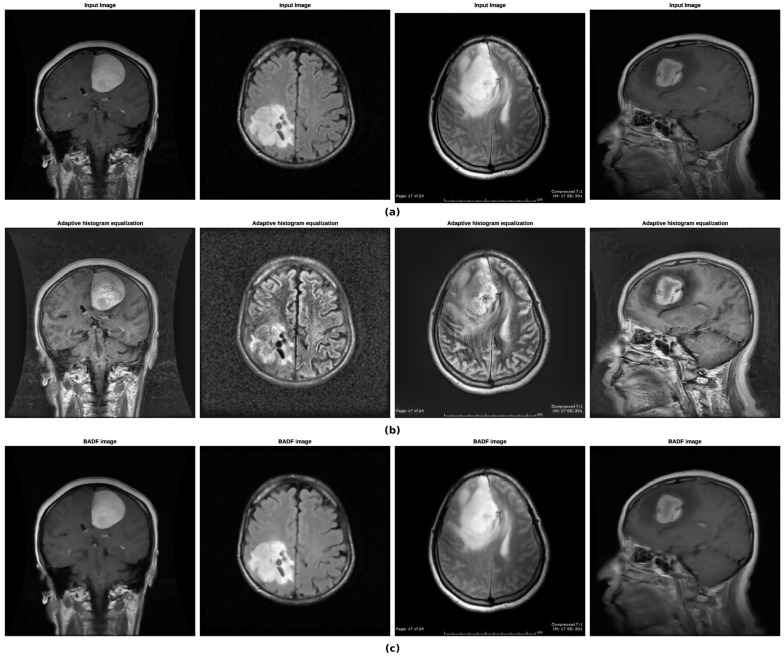
(**a**) Three different grades of input images. (**b**) Noise removal image. (**c**) SMOTE results.

A deep 1D-CNN handles classification, while SMOTE handles the issue of class imbalance. A performance evaluation of the suggested approach for categorizing various imbalanced datasets is also included in this section. There are issues with both oversampling and undersampling. The undersampling algorithms, in particular, remove samples that result in lost information. Overfitting is increased when oversampling generates more samples. For data balancing, the Synthetic Minority Oversampling Technique (SMOTE) is a data-level preprocessing method. The SMOTE methodology is an oversampling method that has demonstrated efficacy in machine learning when used on high-dimensional unbalanced data. To maintain balance in the number of occurrences, the SMOTE approach randomly generates new samples from the minority class. The line that connects the random minority class instance and its closest neighbors is used to create fresh samples. To match the original minority class instances, these instances are constructed using the features of the original data. A balanced dataset is produced by SMOTE and can be utilized for additional classification procedures. Figure 3 shows three different grades of input images: noise-removed images and SMOTE results. Three primary parameters are required by the SMOTE algorithm: the number of nearest neighbors for the minority class, indicated by N; the size of minority class instances; and the degree of oversampling in percentage, represented by M. K represents the number of attributes, while attr represents each attribute. A two-dimensional array called sample [] [] represents the real minority class instances. Prior to the creation of synthetic samples, the indices for the k nearest neighbors for each instance (j) of the minority class are calculated and saved in a variable named nnarray. Synthetic sample production is carried out by the populate() function. It starts by calculating the difference, represented by d, between the feature vector and its closest neighbor. A random number, denoted as rn, is selected between 1 and k. It chooses one of closest k neighbors. By choosing a random number between 0 and 1, represented by g, the gap is found. The artificial samples are tallied and saved in the new index variable, which has a zero initialization. Syn [] [] is a two-dimensional array where the synthetic samples are kept. Because the suggested method requires binary classification on continuous data, SMOTE is preferred in this work. SMOTE is a well-known sampling strategy for class balance when compared to other methods since it expands upon the minority class decision zone.

### 3.3. Segmentation

**Berkeley Wavelet Transform:** One kind of wavelet transform utilized in image processing and analysis is BWT. It is a multiorientation transform with multiple scales that records an image’s directionality and frequency information. Based on the intended frequency and orientation properties, a bank of wavelet filters, also known as filter banks, is created. Usually, a bank of oriented wavelet filters—which are intended to capture various image orientations and scales—is used by the BWT. Usually, Fourier analysis, filter design, and optimization are used in conjunction with other mathematical techniques to create these filters. To acquire wavelet coefficients, the input image is convolved with the wavelet filters at various scales and orientations. To obtain local frequency and directionality information, wavelet filters are usually applied to the image at various scales and locations using a convolution operation. The reaction of the image to the wavelet filters at various scales and orientations is represented by the resulting wavelet coefficients. To extract pertinent features or information from the image, these coefficients can be further examined. The coefficients can be used, for instance, to calculate texture information, edge strength, and other image attributes. Based on eight distinct attributes gathered from the medical photos, four fundamental pairs of wavelets form the foundation of the Berkeley Wavelet Transformation procedures. To extract the individual wavelets and converge them into pairs of wavelets for temporal domain frequencies, each image is sliced at 0, 45, 90, and 125 degrees. Since the individual wavelets are determined to have odd or full symmetries, they are joined in pairs in order to maximize convergence. The entire procedure is efficient, takes little operating time, and yields the best segmentation results because it is based on orthonormal features. Wavelet analysis promises to extract sensitive information and fine-tune the analysis based on the properties examined from the image signals. In the suggested research project, the transformed images from the provided datasets are processed, sent to a gray-level co-occurrence matrix, and used for feature extraction.

(1) $$P=\frac{1}{\sqrt{S}}\;\psi \left(\frac{T-\tau }{S}\right)\;$$

(2) $$\beta_{\theta }^{\phi }\left(\tau ,\;S\right)=\frac{1}{S^{2}}\;\beta_{\theta }^{\phi }\left(3^{S}\left(x-i\right),\;3^{S}\left(y-j\right)\right)$$

Equation (1) represents the scaling function of the Berkeley Wavelet Transform, where the scale and translation parameters enable the multiresolution analysis of the input MRI image. Equation (2) computes the corresponding wavelet coefficients, which extract multiscale spatial and directional features from the MRI image. These enhanced features are subsequently provided as input to the proposed Modified U-Net to improve tumor boundary delineation and segmentation accuracy.

**Figure 4:**
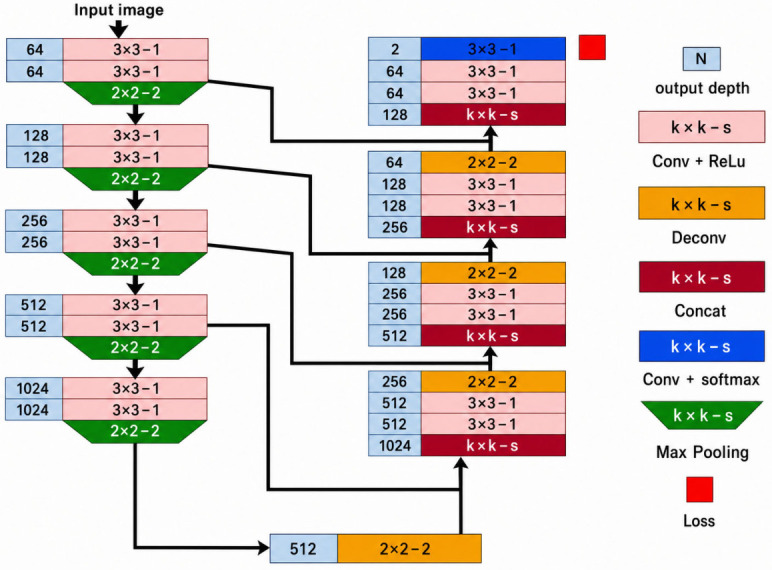
U-Net architecture.

**U-Net:** Semantic segmentation tasks are an area in which the U-Net excels. The U-Net network is widely used in image segmentation for two reasons: first, it can be trained from start to finish and works well with a short dataset; second, there are comparatively few training examples available for U-Net. Figure 4 describes the U-Net’s layer-by-layer design. It provides comprehensive details on the U-Net network at both the contracting path (encoding stage) and the expanding path (decoding stage). As seen in Figure 4’s pink area, the contracting path contains five convolution layers, or a total of ten convolution units, each of which has a *k* × *k* kernel size and s stride before the ReLU activation function. At the encoding stage, a total of five max-pooling layers, k×k filter size and s stride are used, as indicated in green.

The output channel depth at each layer of the U-Net architecture’s encoding and decoding stages is displayed by the gray box. The activation function of ReLU comes after the eight convolution blocks that make up the decoder stage. Its four deconvolution units use the *k* × *k* filter and s stride to deconvolve the input feature image. As seen in mustard yellow, the feature image produced by the deconvolution of the image is half the size of the previous step. The four concatenating units in the decoder step are displayed in burgundy. The convolution block with the softmax activation function, the final block of the decoder stage, is used to classify the feature vector into the probability of the specific class, as indicated by the color blue. The red box represents the loss function, which is used to calculate the error between the target class and the predicted class of the segmented output image. But because U-Net must identify intricate details in the input image, it has a richer structure. However, the suggested techniques must recognize a road feature from a satellite or aerial image, which can only be done with a binary segmentation task. Therefore, a deeper design that requires greater computational memory is not necessary. Additionally, the U-Net model includes many learning parameters that require more training time and slower picture detection speed.

The Berkeley Wavelet Transform (BWT) was incorporated into the proposed framework because of its ability to extract multiscale and multidirectional image features while preserving important structural information. Brain MRI images often contain complex anatomical structures, low-contrast tumor boundaries, and imaging noise, which can affect segmentation performance. BWT decomposes the input MRI image into different frequency components, enhancing edge and texture information while reducing the influence of noise. In the proposed framework, BWT is applied as a preprocessing step before the segmentation stage. The wavelet-enhanced images are then provided as input to the Multilevel Architecture-Based Modified U-Net, allowing the network to learn more representative local and global features for accurate tumor segmentation. This integration improves feature representation and supports a more precise delineation of tumor regions.

### 3.4. The Proposed Multilevel Deep Learning Architecture

The proposed Multilevel Architecture-Based Modified U-Net is designed to provide accurate brain tumor segmentation with reduced architectural complexity. Unlike conventional U-Net and other complex variants that use additional attention, residual, or transformer modules, the proposed model combines imbalance-aware preprocessing with multilevel feature learning and a simplified U-Net structure. The model captures shallow spatial details and deeper semantic tumor features while using fewer encoding and decoding stages. This design aims to improve segmentation accuracy, reduce computational burden, and provide a more efficient framework for MRI-based brain tumor segmentation.

The proposed Multilevel Architecture-Based Modified U-Net follows an encoder–decoder architecture with skip connections that preserve fine spatial information during feature reconstruction. Each encoder block consists of two successive 3 × 3 convolutional layers followed by ReLU activation and a 2 × 2 max-pooling operation for feature extraction and downsampling. The decoder employs transposed convolution for upsampling, followed by concatenation with the corresponding encoder features through skip connections to recover spatial details. Unlike the conventional U-Net, the proposed model reduces the encoder depth from five downsampling stages to three and simplifies the decoder accordingly, resulting in fewer convolutional blocks and trainable parameters. A softmax activation function is applied in the final layer to generate pixel-wise segmentation probabilities, while the network is optimized using the cross-entropy loss function. This lightweight design reduces computational complexity while preserving multilevel feature learning for accurate brain tumor segmentation.

The proposed deep learning framework in the suggested model has little to no variation between the layers, which are typically referred to as shallow and deep layers with distinct functions. The architecture’s layers are intricately interconnected, enabling efficient communication via a knowledge or information bridge that operates on the local and global characteristics found in the input images.

**Figure 5:**
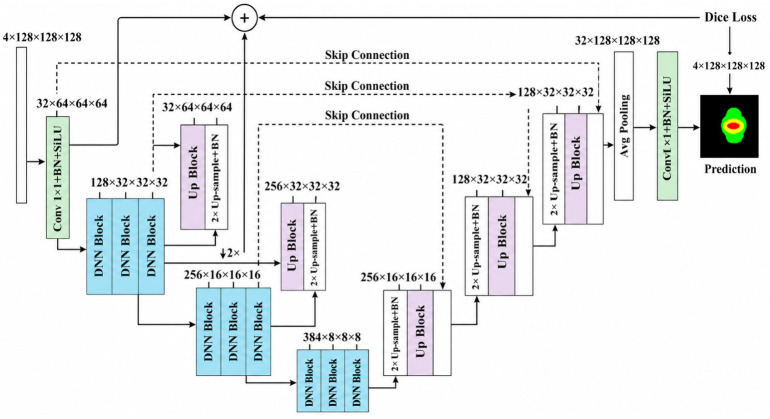
Proposed multilevel deep learning-based architecture.

The architecture of the suggested model is shown in Figure 5, and when 128 × 128 images are input into the model, correct segmentation results are produced. The segmentation results are generated in the same dimensions after the photos are analyzed. To ensure promising reconstruction outcomes, the features are further refined and classified using a multilevel learning technique once the downsampling procedure is completed. Multilevel learning architectures are frequently employed in the context of image processing for tasks like object detection, picture segmentation, and image recognition, where the input images may contain features or objects of different sizes or scales. The ability of these architectures to incorporate various processing levels allows them to collect features at varying spatial resolutions, as well as local details and global context, which improves efficiency while handling objects or features with varying sizes or scales. An image or a collection of images of brain tumors is typically used as input by the suggested architecture. Certain deep learning architectures for image segmentation may accept extra input in addition to the input photos, such as image metadata, patient demographics, or other contextual data, in order to guide the process or enhance the accuracy of segmentation [19].

The proposed architecture shown in Figure 5 keeps a decoder and an encoder in addition to a few layers for padding. The encoder’s convolutional layers are in charge of producing the best possible pooling outcomes, and the dropout layers make sure that this goal is met. The encoder currently uses two convolutional layers, feeding the output to the next levels to perform a Conv2D Transpose operation. Before the decoder is processed, all of these procedures are finished. Each layer’s encoding and decoding stages were altered in the revised U-Net. As seen in Figure 5, the proposed architecture’s four upsample parts were reduced to three, while its five downsample parts were reduced to two. As a result, the updated U-Net’s encoding step uses three max-pooling and seven convolution units rather than the ten convolution units and five max-pooling layers of the original U-Net architecture. The three components make up the improved U-Net’s decoding step.

**Figure 6:**
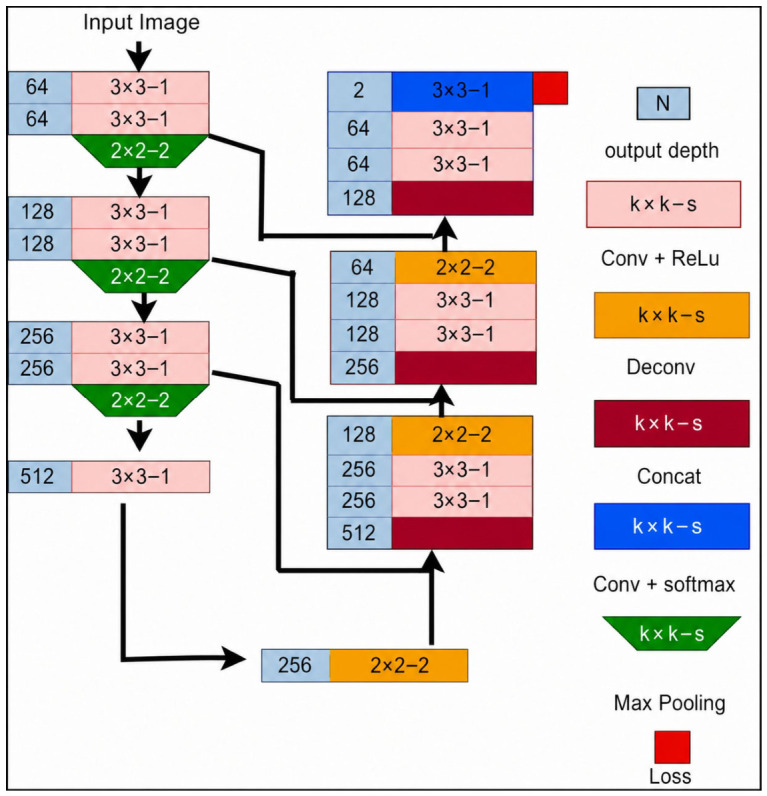
Modified U-Net.

As shown in Figure 6, each unit consists of one concat unit, two repeating convolution units, and one deconvolution unit. Nevertheless, the six convolution functions, three concat functions, and two deconvolution units make up the decoder stage of the U-Net architecture. In order to detect the road network using Modified U-Net, a total of 13 convolution blocks with a ReLU activation function, three max-pooling layers, three concat blocks, three deconvolution blocks, and one convolution block with a softmax activation function are needed. A softmax layer is affixed to the decoder network’s last stage, converting the output into probability maps. The road-detecting network in this model is trained using cross-entropy loss, which is described as

(3) $$L_{bce}=\sum_{i-1}^{b}\sum_{j-1}^{n}\left[\;{GT}_{ij}\log\left(pred_{ij}\right)+\left(1-{GT}_{ij}\right)\log\left(1-pred_{ij}\right)\right]$$

Equation (3) defines the binary cross-entropy (BCE) loss function used to optimize the proposed Multilevel Architecture-Based Modified U-Net during training. The loss function measures the discrepancy between the predicted segmentation probability and the corresponding ground truth label for each pixel in the MRI image. Here, *GT_ij_* denotes the ground truth label of the *j*-th pixel in the *i*-th image batch, while *pred_ij_* represents the predicted probability generated by the network for the corresponding pixel. The binary cross-entropy loss penalizes incorrect pixel-wise predictions and encourages the network to produce segmentation masks that closely match the expert-annotated ground truth images. Minimizing this loss during training enables the model to learn accurate tumor boundaries and improve overall segmentation performance. The sigmoid function is applied to the weighted sums of the hidden layer activation functions to generate the outputs of the modified U-Net model.

(4) $${pred}_{i}=\frac{1}{1+e^{-\theta_{i}}}$$

(5) $$\theta_{i}=\sum_{j=1}^{l}w_{ij}h_{j}$$

Equations (4) and (5) describe the prediction process of the proposed Modified U-Net. Equation (4) applies the sigmoid activation function to convert the weighted input into a probability value between 0 and 1, representing the likelihood that a pixel belongs to the tumor region. Equation (5) computes the weighted input as the linear combination of the input features and their corresponding learnable weights. Together, these equations enable pixel-wise probability estimation, which is subsequently used by the binary cross-entropy (BCE) loss function to optimize the network for accurate brain tumor segmentation. We may determine the derivative of the error for each weight connecting the hidden and output units by applying the chain rule,

(6) $$\frac{{\partial L}_{bce}}{{\partial w}_{ij}}=\frac{{\partial L}_{bce}}{{\partial pred}_{ij}}\;\frac{{\partial L}_{ij}\;}{{\partial 0}_{ij}}\;\frac{{\partial 0}_{ij}}{{\partial w}_{ij}}$$

**Figure 7:**
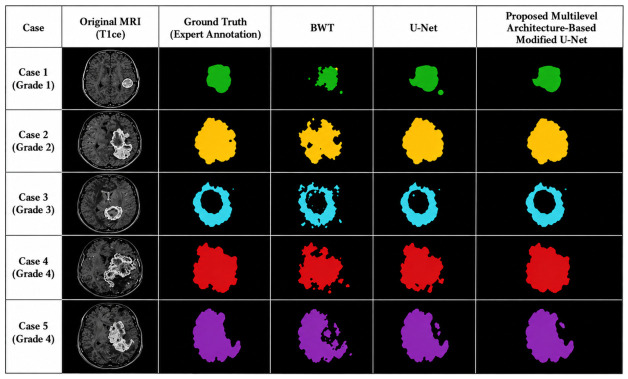
Qualitative comparison of brain tumor segmentation.

Equation (6) represents the gradient computation of the binary cross-entropy (BCE) loss function with respect to network weights during backpropagation. Using the chain rule, the gradient of the loss with respect to each weight *w_ij_* is calculated by combining the derivatives of the loss function, the predicted output, and the neuron’s weighted input. This gradient is used to iteratively update the learnable weights, enabling the proposed Multilevel Architecture-Based Modified U-Net to minimize the segmentation error and improve the accuracy of brain tumor segmentation during training. Figure 7 shows a qualitative comparison of the brain tumor segmentation results. This figure presents a qualitative comparison between the proposed method and the reference segmentation approaches. Compared with BWT and the conventional U-Net, the proposed Multilevel Architecture-Based Modified U-Net generates segmentation masks that more closely resemble the expert-annotated ground truth masks. In particular, the proposed model provides improved delineation of tumor boundaries while reducing under-segmentation and over-segmentation in complex tumor regions. The proposed method shows closer agreement with the expert-annotated ground truth mask and provides a more accurate delineation of tumor boundaries.

## 4. Simulation Results and Discussion

This section discusses the segmentation process analysis that was conducted using the Modified U-Net approach, which is based on a multilevel architecture.

### Experimental Setup

The proposed Multilevel Architecture-Based Modified U-Net was implemented in MATLAB using the Deep Learning Toolbox. Prior to training, all MRI images were resized to 128 × 128 pixels. The network was trained using the Adam optimizer with an initial learning rate of 0.001, a batch size of 16, and 100 epochs. Cross-entropy loss was used as the objective function. ReLU activation was employed in all convolutional layers, while the final segmentation layer used the softmax activation function to generate pixel-wise probability maps. All experiments were performed on a CPU-based workstation using MATLAB R2026a, without GPU acceleration. Implementation was intended to evaluate the effectiveness of the proposed segmentation framework rather than to optimize computational performance.

The MRI input pictures were taken from the BraTS collection. Both benign and malignant brain tumor pictures are present. SMOTE works successfully even with extremely unbalanced TCIA datasets. The standardization technique scales each feature before applying SMOTE. The grade 3 and 4 datasets, which are low-imbalanced datasets, are used to create synthetic samples that are visualized.

**Figure 8:**
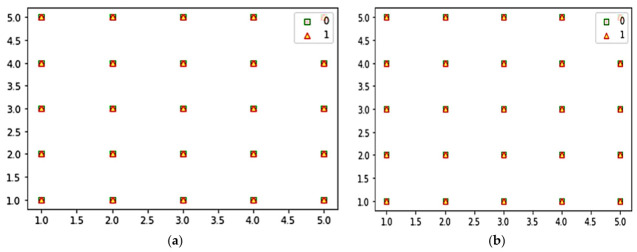
Balance scale dataset. (**a**) Original data. (**b**) Class-balanced data.

Figure 8a,b display the balanced data from the various datasets as well as the class distribution from the original dataset.

**Figure 9:**
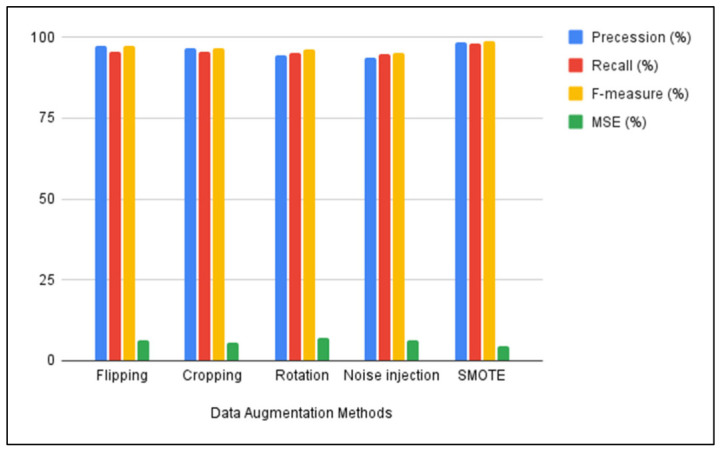
Comparison of various augmentation techniques.

Figure 9 illustrates how various augmentation techniques work using various parameter metrics. In this examination, the performance of the total augmentation technique was superior to that of the individual techniques.

Table 1 presents the segmentation performance of BWT, conventional U-Net, and the proposed Multilevel Architecture-Based Modified U-Net across four tumor grades. The proposed model achieved higher Dice coefficient, Jaccard coefficient, and MCC values than the comparison methods in all grades, indicating improved tumor region overlap and stronger agreement with the ground truth masks. Although the accuracy values are also high, accuracy should be interpreted cautiously because brain tumor segmentation is a highly imbalanced task in which background pixels dominate the image. Therefore, the Dice coefficient, Jaccard coefficient, and MCC were considered more informative indicators of segmentation performance. The improved results suggest that the proposed model provides more consistent tumor delineation than BWT and conventional U-Net, particularly for grades 1 and 4, where the performance gap is more evident.

Table 1 reports the average segmentation performance for each tumor grade. Although the proposed model achieved an improved Dice coefficient, Jaccard coefficient, and MCC compared with BWT and conventional U-Net, the current table does not include standard deviation or confidence intervals because only aggregated evaluation values were available. This is acknowledged as a limitation of the present study. In future work, per-patient segmentation results will be reported as the mean ± standard deviation, together with confidence intervals and boxplot visualizations, to provide a more detailed assessment of model robustness and statistical variability.

**Figure 10:**
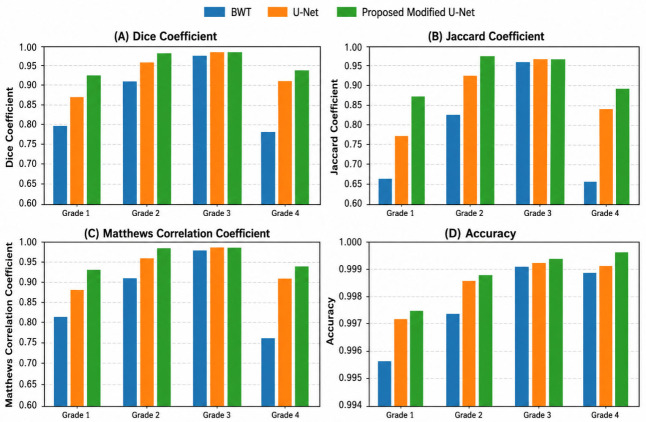
Comparison of classification accuracy with SMOTE augmentation.

Figure 10 presents a comparative analysis of the segmentation performance of the BWT, conventional U-Net, and the proposed Multilevel Architecture-Based Modified U-Net across four brain tumor grades using the Dice coefficient, Jaccard coefficient, Matthews correlation coefficient (MCC), and Accuracy metrics. As shown in Figure 10A, the proposed model consistently achieved higher Dice coefficients than the comparison methods, indicating improved overlap between the predicted segmentation and the ground truth tumor regions. Similar improvements are observed in Figure 10B, where the proposed model attained higher Jaccard coefficients, demonstrating more accurate delineation of tumor boundaries. Figure 10C shows that the proposed approach also achieved the highest MCC values across all tumor grades, indicating stronger agreement between predicted segmentation and the reference annotations. Although all three methods produced high accuracy values, as illustrated in Figure 10D, the differences among the models are relatively small because accuracy is less sensitive to class imbalance in medical image segmentation. Overall, the graphical comparison demonstrates that the proposed Multilevel Architecture-Based Modified U-Net consistently outperformed the BWT and conventional U-Net models, with particularly notable improvements for grade 1 and grade 4 tumors, highlighting its effectiveness in segmenting tumors with varying morphological characteristics.

## 5. Discussion

Recent medical image segmentation models have significantly extended the original U-Net architecture. Attention U-Net incorporates attention gates to improve localization by suppressing irrelevant image regions [20]. U-Net++ employs nested and dense skip connections to reduce the semantic gap between encoder and decoder feature maps [21]. nnU-Net introduced a self-configuring framework that automatically adapts preprocessing, network architecture, and training strategies for biomedical image segmentation [22]. More recently, transformer-based architectures, such as TransUNet and Swin-Unet, have demonstrated superior capability in capturing long-range contextual information while preserving local image features [23,24]. In addition, Swin UNETR has shown competitive performance for brain tumor segmentation on the BraTS benchmark by integrating a hierarchical Swin Transformer encoder with a CNN-based decoder [25]. Compared with these advanced models, the proposed Multilevel Architecture-Based Modified U-Net focuses on a lightweight segmentation framework that combines imbalance-aware preprocessing, multilevel feature learning, and a simplified encoder–decoder architecture. Although the present study experimentally compares the proposed model with the conventional U-Net and BWT under identical implementation settings, a comprehensive experimental comparison with these recent architectures will be considered in future work.

Although the proposed model achieved high pixel-wise accuracy, accuracy alone is not sufficient for evaluating brain tumor segmentation because MRI images contain a large background region and relatively small tumor regions. Therefore, high accuracy may be obtained even when tumor boundaries are not accurately segmented. For this reason, greater emphasis was placed on the Dice coefficient, Jaccard coefficient, and MCC, which provide a more meaningful assessment of segmentation quality under class-imbalanced conditions. In the present results, the proposed Multilevel Architecture-Based Modified U-Net showed improved Dice and Jaccard values compared with BWT and conventional U-Net, indicating better tumor region overlap with the ground truth masks. Future work will further include sensitivity, specificity, and Hausdorff distance to provide a more complete evaluation of boundary accuracy and clinical segmentation reliability.

A limitation of this study is that the proposed model was evaluated only on the BraTS dataset. Although BraTS is a widely used benchmark dataset for brain tumor segmentation, it may not fully represent the variability observed in real clinical MRI data, including differences in scanners, imaging protocols, image quality, tumor appearance, and patient populations. Therefore, the generalizability of the proposed method to independent clinical settings remains to be fully established. Future work will include external validation using multicenter clinical datasets to assess the robustness and clinical applicability of the proposed framework.

The proposed Multilevel Architecture-Based Modified U-Net employs a simplified encoder–decoder architecture with fewer encoding and decoding stages than the conventional U-Net, thereby reducing the number of trainable layers and computational operations. Although this architectural simplification is expected to improve computational efficiency, the present study did not include the quantitative benchmarking of training time, inference time, GPU memory consumption, or computational complexity (e.g., FLOPs). Therefore, the computational advantage should be interpreted as an architectural benefit rather than an experimentally verified performance gain. Future work will include a comprehensive computational analysis by reporting training time, inference speed, memory usage, and hardware specifications to provide a complete assessment of the proposed model’s efficiency.

## 6. Conclusions

This study proposed an automated brain tumor segmentation framework using MRI images and a Multilevel Architecture-Based Modified U-Net. The proposed method is intended to support tumor region delineation in MRI scans and should be regarded as a computer-aided segmentation tool rather than a standalone diagnostic system. The proposed method integrates preprocessing, class imbalance handling using modified SMOTE, and a lightweight Modified U-Net architecture to improve tumor region detection. The experimental results on the BraTS dataset showed that the proposed model achieved a better Dice coefficient, Jaccard coefficient, MCC, and accuracy than BWT and conventional U-Net across different tumor grades. These findings indicate that the proposed framework can provide more accurate and consistent tumor segmentation while reducing model complexity. Despite these promising results, this study has some limitations. The model was evaluated only on the BraTS dataset, and external validation using independent clinical datasets was not performed. In addition, standard deviation, confidence intervals, Hausdorff distance, sensitivity, and specificity were not included in the current evaluation. Future work will focus on validating the proposed model on multicenter clinical MRI datasets, comparing it with recent architectures such as Attention U-Net, U-Net++, nnU-Net, TransUNet, and Swin-Unet and reporting more comprehensive statistical and boundary-based evaluation metrics.

## Figures and Tables

**Table 1:** Performance evaluation of proposed segmentation models.

	Metrics	BWT	U-Net	Multilevel Architecture-Based Modified U-Net
**Grade 1**	*Dice Coefficient*	0.7964	0.8714	0.9326
	*Jaccard Coefficient*	0.6617	0.7721	0.8737
	*MCC*	0.8117	0.8775	0.9340
	*Accuracy (%)*	0.9957	0.9972	0.9975
**Grade 2**	*Dice Coefficient*	0.9088	0.9616	0.9884
	*Jaccard Coefficient*	0.8328	0.9260	0.9770
	*MCC*	0.9114	0.9617	0.9882
	*Accuracy*	0.9974	0.9986	0.9988
**Grade 3**	*Dice Coefficient*	0.9818	0.9863	0.9868
	*Jaccard Coefficient*	0.9642	0.9729	0.9740
	*MCC*	0.9814	0.9859	0.9864
	*Accuracy*	0.9991	0.9992	0.9993
**Grade 4**	*Dice Coefficient*	0.7846	0.9133	0.9442
	*Jaccard Coefficient*	0.6456	0.8405	0.8943
	*MCC*	0.7647	0.9163	0.9443
	*Accuracy*	0.9989	0.9991	0.9996

## Data Availability

This study utilized the publicly available Brain Tumor Segmentation (BraTS) dataset provided by the Medical Image Computing and Computer Assisted Intervention (MICCAI) challenge. The dataset comprises multimodal magnetic resonance imaging (MRI) scans (T1, T1ce, T2, and FLAIR) with expert-annotated ground truth segmentations of brain tumors. The dataset can be accessed upon registration from the official BraTS challenge website https://www.med.upenn.edu/cbica/brats2020/data.html (accessed on 2 June 2026).
